# *Staphylococcus aureus* bacteremia with a mediastinal abscess in a 9-month-old infant: a case report and literature review

**DOI:** 10.11604/pamj.2023.44.173.38638

**Published:** 2023-04-13

**Authors:** Jouhadi Zineb, Chrislaine Moboula, Nassid Meryem, Fatene Abdallah, Chbani Kamilia, Boubia Souhail

**Affiliations:** 1 Department of Pediatric Infectious Diseases, Children’s Hospital, CHU Ibn Rochd Medical School, Hassan II University, Casablanca, Morocco,; 2 Department of Thoracic Surgery, CHU Ibn Rochd Medical School, Hassan II University, Casablanca, Morocco,; 3 Department of Pediatric Radiology, Children´s Hospital, CHU Ibn Rochd Medical School, Has san II University, Casablanca, Morocco

**Keywords:** Mediastinal abscess, *Staphylococcus aureus* bacteremia, case report

## Abstract

Non-traumatic mediastinal abscesses are very rare in children; we can classify them into 2 types: descending mediastinitis (or mediastinitis by extension or by contiguity) complicating an otorhinolaryngological or esophageal etiology and mediastinitis generated by direct blood inoculation in a context of a septicemia or primary mediastinitis which is exceptional. We describe a case of right pleuropulmonary staphylococcal disease with bilateral mediastinal localization in a previously healthy 9-month-old infant. It was revealed by sepsis with severe respiratory distress. The germ was isolated from the pleural puncture fluid. A thoracic computed tomography was indicated due to a widening mediastinum noted on chest X-ray in addition to pleuropulmonary involvement. Thoracic computed tomography revealed a huge bilateral mediastinal abscess which was curbed thanks to right pleural drainage with adapted antibiotic therapy. Other investigations did not show any immune abnormalities in this infant. Mediastinitis represents a diagnostic and therapeutic emergency; those that are secondary to direct blood or lymphatic dissemination even very rare; should be considered in any context of severe sepsis including staphylococcus or streptococcus pneumonia. Since 1985 only 11 cases of such mediastinal abscesses have been reported.

## Introduction

Mediastinal abscesses of non-traumatic etiology are relatively rare in children. There are two types of nontraumatic mediastinitis: the first type and the most known is mediastinitis by extension or by contiguity in which; the infection spreads along the peri-visceral cervical-mediastinal fasciae; they are named: acute descending mediastinitis always complicating an otolaryngologic or esophageal etiology. The second type is the primary mediastinal abscess resulting from blood or lymphatic dissemination in the context of sepsis, which is otherwise exceptional. In both cases, early diagnosis with appropriate antibiotic therapy and percutaneous or surgical draining is essential for effective management. We report a case of a right staphylococcal pleuropulmonary disease with a mediastinal localization in an infant.

## Patient and observation

**Patient information:** a 9-month-old previously healthy girl, from a non-consanguineous parent, was admitted to the infectious pediatric department for severe febrile respiratory distress. This infant does not have any history of recent otorhinolaryngological or skin infections. Her illness dated back to eight days before admission with an isolated fever of 39-40°C, which justified a consultation, and the infant was put on oral amoxicillin and antipyretics. Her clinical condition worsened by the occurrence of severe respiratory distress which necessitated her emergency hospitalization.

**Clinical findings:** the admission examination found a conscious infant, eutrophic at 9 kg with a normal height of 77 cm. Vital signs revealed a temperature of 39.5°C, respiratory rate of 60 breaths/minute, heart rate of 110 beats/minute, her pulse oximetry was 86% on ambient air (93% under oxygen), perioral cyanosis with signs of respiratory distress such as the flapping of the wings of the nose, with severe subcostal and suprasternal retractions, pleuropulmonary examination showed a decrease in vesicular murmurs on the right lung.

**Figure 1:**
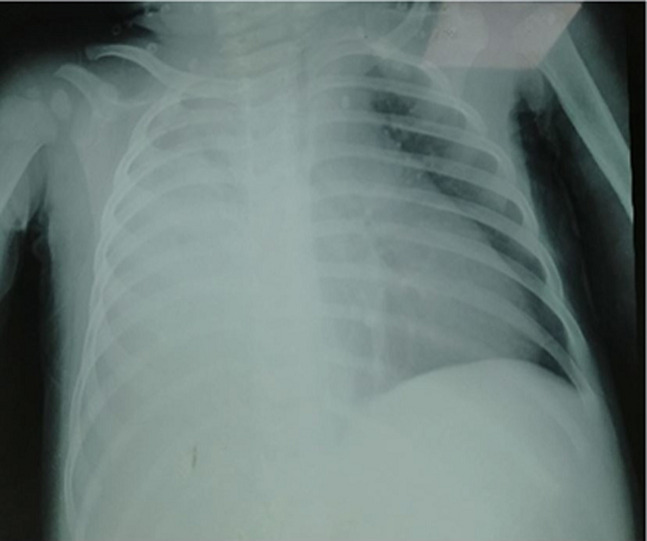
the initial chest X-ray showing an effusion and pneumonia on the right with a mediastinal widening and a displacement of the heart to the left

**Figure 2:**
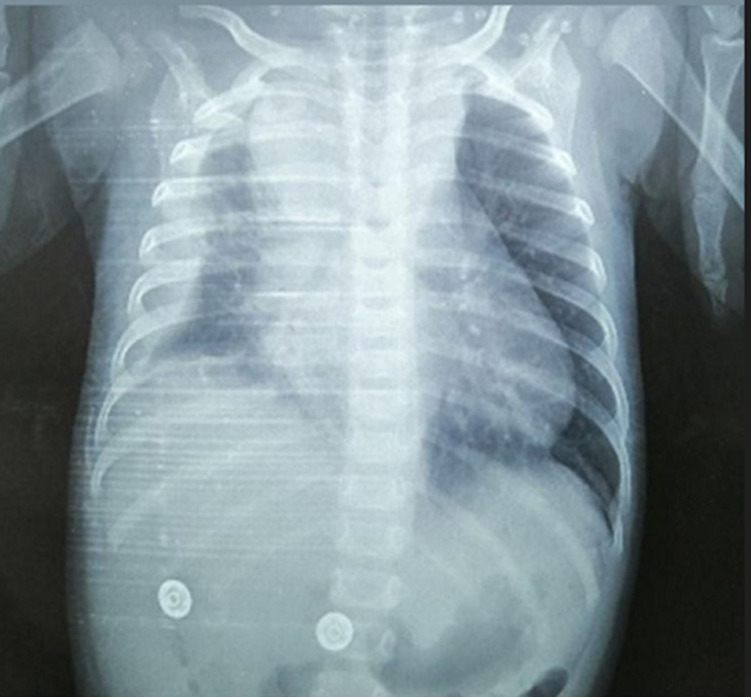
the control chest X-ray after evacuating puncture showing right loculated pleurisy and mediastinal enlargement with a heart pushed back to the left

**Diagnostic assessment:** the chest X-ray confirmed the clinical data by highlighting an effusion and pneumonia on the right with a mediastinal widening and a displacement of the heart to the left (Figure 1). A chest ultrasound evaluation confirmed a large right pleural effusion. The child got an evacuating puncture bringing back 200 ml of the purulent pleural fluid; with uncountable cells including 80% of neutrophils and proteins at 62 g/l. Later, the culture grew a methicillin-sensitive Staphylococcus aureus. At the biological assessment, the blood culture was sterile, and the C-Reactive Protein (CRP) was at 320 mg/l. Blood count revealed microcytic hypochromic anemia at 9.1 g/dl, hyperleukocytosis at 19100 /mm^3^ predominantly polynuclear neutrophils at 12190 /mm^3^, lymphocytes at 4470 /mm^3^ and thrombocytosis at 874 000 /mm^3^. Human immunodeficiency virus serology was negative. The course was marked by worsening respiratory distress with a control chest X-ray showing right loculated pleurisy and mediastinal enlargement with a heart pushed back to the left (Figure 2).

**Figure 3:**
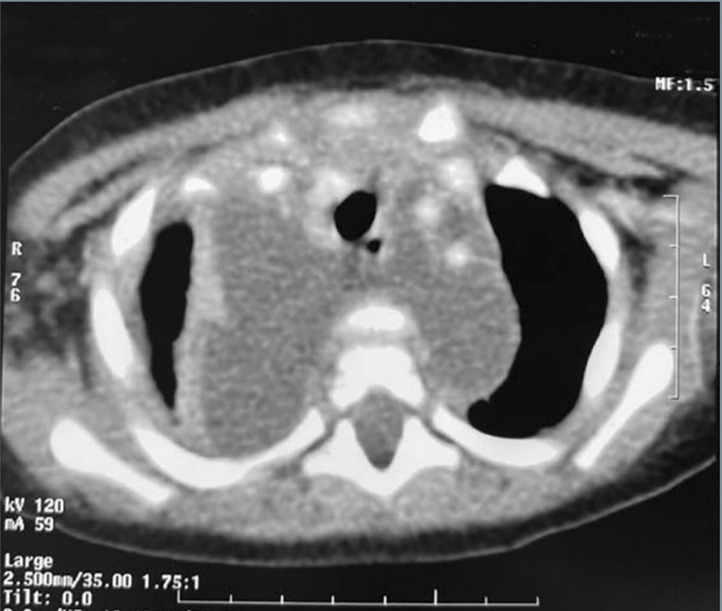
a thoracic CT scan showing a collected diffuse mediastinitis spread to the cervical region associated with a right pleural empyema and pulmonary condensation

**Diagnosis:** thoracic computed tomography was performed showing a collected diffuse mediastinitis spread to the cervical region associated with a right pleural empyema and pulmonary condensation (Figure 3).

**Figure 4:**
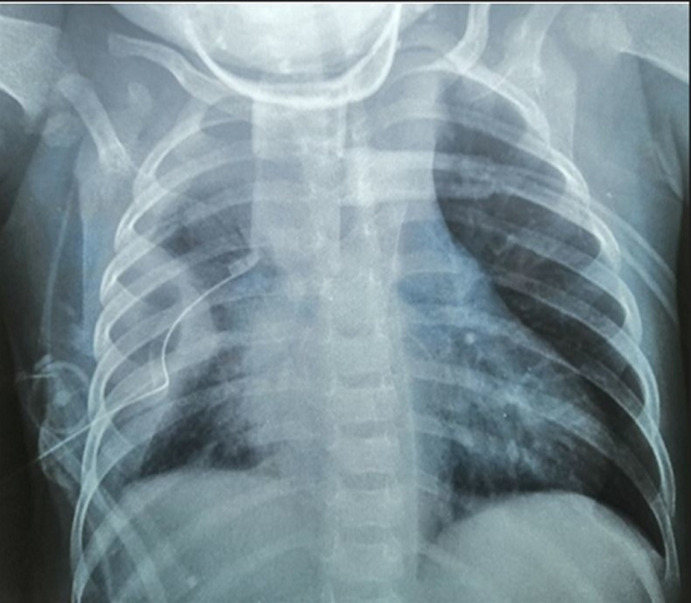
the immediate chest X-ray control showing a mediastinal enlargement and a right septate pleurisy with a drain well-placed

**Figure 5:**
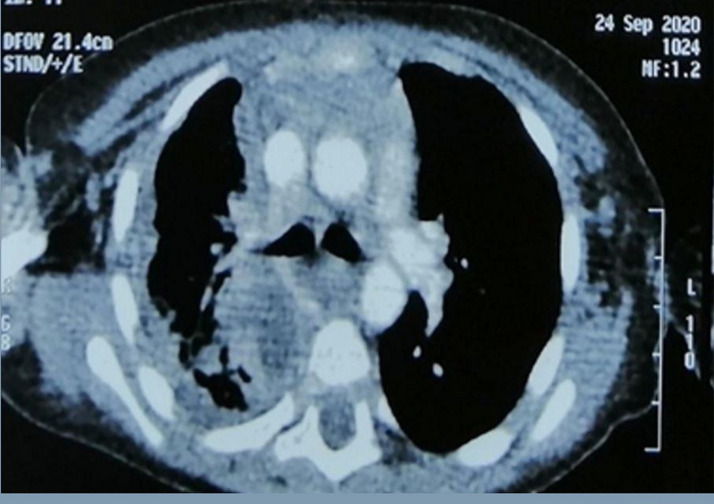
the control thoracic CT scan showing regression of the mediastinal collection and of the pleural empyema

**Therapeutic interventions:** therapeutically, the infant received Ceftriaxone 150 mg/kg/day, Gentamycin 3 mg/kg/day, and metronidazole 30 mg/kg/day. A chest percutaneous drainage brought back 600 ml of pus; the immediate chest X-ray control showed a right septate pleurisy with a drain well placed (Figure 4). The evolution was favorable after 10 days of thoracic drainage and antibiotic treatment of 3 weeks. A control thoracic computed tomography scan was performed showing regression of the mediastinal collection and of the pleural empyema (Figure 5). The biological evaluation just before the patient was discharged was very comforting with leukocytes at 12400 /mm^3^, polynuclear neutrophils at 7240 /mm^3^and platelets at 596,000 /mm^3^, CRP at 47.7 mg/l.

**Follow-up and outcome:** the follow-up is 1 year without any sequelae.

**Informed consent:** there was no need for informed consent because all investigations were done as part of a classic management of any sepsis state in infants.

## Discussion

Mediastinal abscesses secondary to hematogenous and/or lymphatic spread from distant infection sites have been rarely described comparatively with descending mediastinitis complicating otolaryngologic or esophageal infectious sites; which spreads along the peri-visceral cervical-mediastinal fasciaes. Therefore, mediastinal abscesses as a secondary localization of Staphylococcus aureus bacteremia in children are extremely rare; only 11 cases have been reported in the literature since 1985 (Table 1). The mean age of these cases was 4 years with extremes of 15 days to 11 years old, eight cases were related to *Staphylococcus aureus*, and three cases were of pneumococcal origin [1,2]. The starting point of sepsis for the literature´s eight cases was known in only 3/5 cases: a cutaneous infection in 2 cases and a veinitis in 1 case [3,4]. In the remaining 5 other cases as also our case the origin of SAB was not determined.

**Table 1:** clinical features of patients with primary mediastinal abscess

First author	Country	Date of publication	Age	Gender	Title of the publication	germ	Starting point	Other organ localisation	Evolution
Lotan C,	Israël	1985	16 months	F	Non-traumatic pneumococcal mediastinal abscess	*Streptococcus pneumonia*	Pneumonia	-	Favorable
Tobias JD	USA	1990	8 years	F	Pneumococcal mediastinal abscess	*Streptococcus pneumonia*	Right pneumonia	pleural effusion	Favorable
Smith A	Zimbabwe	1992	11 years	M	Anterior mediastinal abscess	*Staphylococcus aureus*	-	Septic arthritis - pericardial tamponnade	Favorable
Bungay HK	UK	1995	6 weeks	F	Staphylococcal anterior mediastinal abscess	*Staphylococcus aureus*	cannula site	-	Favorable
Fields JM	USA	1997	22 months	M	Idiopathic bilateral anterior mediastinal abscesses	*Streptococcus pneumonia*	-	-	Favorable
Vera L. J.Krebs	Brazil	2000	15 days	F	Anterior mediastinal abscess	*Staphylococcus aureus*	Right Pneumonia Infraclavicular Subcutaneous abscess	Right pleural effusion Septic arthritis	Favorable
S Tercier	Switzerland	2005	12 months	M	Mediastinal abscess	*Staphylococcus aureus*	Left axillary Abscesses	Pericarditis Left pleural effusion	Favorable
Gamiao JA	Philippine	2008	5 years	M	Anterior mediastinal abscess	*Staphylococcus aureus*	Pneumonia	Pleural effusion Myocarditis Pericardial effusion Endocarditis	Favorable
Kumar S	Inde	2011	5 years	M	Non-traumatic anterior mediastinal abscess	*Staphylococcus aureus*	Right thigh abscess Left axillary abscess	Right pneumonia Pleural empyema Pericardial effusion	Favorable
Hernandez LE	USA	2013	10 years	M	Anterior mediastinal abscess	*Staphylococcus aureus*	-	Bilateral pleural effusions (reactional) Sternum osteomyelitis	Favorable
Sanchez J	New york	2018	8 months	F	Community-Acquired MRSA Pericarditis and Mediastinitis	*Staphylococcus aureus*-MRSA	-	Pericardial effusion	Favorable
Zineb J (Our case)	Morocco	2021	9 months	F	*Staphylococcus aureus* bacteremia and mediastinal abscess	*Staphylococcus aureus*	Right Pneumonia	Pleural effusion	Favorable

MRSA: methicillin-resistant *Staphylococcus aureus* F: female M: male

Different septic localizations associated with mediastinal abscess were pleural empyema in 5 cases [2,5-7] pericarditis in 6 cases [3,8-10], arthritis in 2 cases [6,9] sternum osteomyelitis in 1 case [8] and endocarditis in 1 case [7]. The most common causative organism identified in the primary mediastinal abscess is methicillin-sensitive *Staphylococcus aureus* in all cases. However, cases, caused by *Streptococcus pneumoniae* and mixed aerobic and anaerobic bacteria, have also been reported [1,2,5,11].

A chest X-ray may suggest the existence of a mediastinal abscess by highlighting a mediastinal widening. Contrast-enhanced computed tomography scan of the neck and thorax is the imaging modality of choice which gives information regarding the extent of the spread of the abscess, and its relationship with vital organs; guiding, therefore, the physician to decide the approach for draining the abscess [8]. Early diagnosis and treatment are important to decrease the mortality and morbidity related to SAB with mediastinal abscess. Appropriate antibiotics along with a minimal invasive percutaneous drainage are a good option for sustained healing.

## Conclusion

*Staphylococcus aureus* bacteremia is a life-threatening infection in pediatric patients specifically in the first months of their life; septic localization related to this bacteremia could be anywhere in the body. among others, the mediastinal abscesses should be actively researched as well as pericarditis, endocarditis, and osteoarticular localizations.

## References

[1] Lotan C, Boneh A, Tamir I, Goitein KJ (1985). Case reports An unusual case of non-traumatic pneumococcal mediastinal abscess. Intensive Care Med.

[2] Tobias JD, Bozeman PM (1990). Pneumococcal abscess presenting as an anterior mediastinal mass in an eight-year-old child. Pediatr Infect Dis J.

[3] Tercier S, Vasseur-Maurer S, Matoso V, Hohlfeld J, Joseph JM (2005). Huge mediastinal abscess in a 12-month-old child: case report and review of the literature.

[4] Bungay HK, Shefler AG, McHugh K (1995). CT of staphylococcal anterior mediastinal abscess in an infant. Pediatr Radiol.

[5] Kumar S, Kumar V, Bishnoi A, Chadha R (2011). Non-traumatic anterior mediastinal abscess in childhood. J Indian Assoc Pediatr Surg.

[6] Krebs VL, Tenório PB, Valente M, de Diniz EM, Ceccon ME, Vaz FA (2000). Computed tomography of anterior mediastinal abscess in a neonate. Pediatr Radiol.

[7] Hernandez LE, Shepard C, Hoggard E, Bryant RI, Hernandez LE, Shepard C, Hoggard E, Bryant III R (2013). Isolated Staphylococcal anterior mediastinal abscess in a 10-year-old-boy with chest pain and fever. Isolated Staphylococcal anterior mediastinal abscess in a 10-year-old-boy with chest pain and fever. J Pediatr Infect Dis.

[8] Gamiao JA (2008). A Huge Anterior Mediastinal Abscess In A 5-year-old Male: A Case Report. Pediatr Pulmonology-Case Rep.

[9] Smith A, Sinzobahamvya N (1992). Anterior mediastinal abscess complicating septic arthritis. J Pediatr Surg.

[10] Sanchez J, Schneider A, Tretter J, Shopsin B, Al-Qaqaa Y, Khaitan A (2017). Community-Acquired MRSA Pericarditis and Mediastinitis in a Previously Healthy Infant. J Pediatr Intensive Care.

[11] Fields JM, Schwartz DS, Gosche J, Keller MS (1997). Idiopathic bilateral anterior mediastinal abscesses. Pediatr Radiol.

